# Fertility desire and associated factors among clients on highly active antiretroviral treatment at finoteselam hospital Northwest Ethiopia: a cross sectional study

**DOI:** 10.1186/s12978-015-0063-2

**Published:** 2015-08-11

**Authors:** Fassika Abbawa, Worku Awoke, Yayehirad Alemu

**Affiliations:** Gamby College of Medical Sciences, BahirDar, Ethiopia; Department of Epidemiology, School of Public Health, College of Medicine and Health Sciences, BahirDar University, BahirDar, Ethiopia; Department of Public Health, College of Health Science, Mizan-Tepi University, MizanTeferi, Ethiopia

**Keywords:** HAART, Fertility desire, Ethiopia

## Abstract

**Background:**

The accessibility of antiretroviral treatment changed the lives of persons living with HIV from hopelessness to hopefulness. Thus, many of them decided to have children. In Ethiopia, where there is high prevalence of HIV, level of fertility desire among persons living with HIV could have significant part in safe motherhood and child health. The aim of this study was to assess the level of fertility desire and identify factors associated with it among clients on highly active antiretroviral treatment at Finoteselam Hospital, Northwest Ethiopia.

**Methods:**

A cross-sectional study design supplemented by in-depth interview was conducted on 422 clients on Highly Active Antiretroviral Treatment from July 1 to August 12, 2013. Structured questionnaire was used to collect the data. Data were entered in to EPi Info version 3.5.1 and exported to SPSS software version 16 for further analysis. Descriptive and summary statistics were computed. Proportions were calculated to estimate fertility desire level. Binary logistic regression model was fitted to identify factors associated with fertility desire.

**Results:**

A total of 422 clients were included in the study of which 217 (51.4 %) were males. The median age was 33 (IQR = 12) years. A total of 141(33.4 %) of clients had desire for having children. Male clients desire children than their female counterparts [AOR = 3.19, 95 % CI: (1.56, 6.51)]. Clients who had no child had more desire for having children than those who had three or more children [AOR = 6.78, 95 % CI: (2.38, 19.27)] and those who had ≤2 years duration on ART had more desire than those with >2 years duration on ART [AOR = 3.64, 95 % CI: (1.74, 7.64)]. Clients who had discussion with ART service provider about sexuality, Fertility desire and family planning had more child desire [AOR = 3.12, 95 % CI: (1.54, 6.32)].

**Conclusions:**

One third of clients have desire to have a child/children in the future. Male clients and clients who have less than or equal to 2 years ART follow up, with no child and having discussion with ART service provider were associated with increased fertility desire. Guidelines formulated and counseling protocols developed shall consider this desire to achieve their reproductive goals in the healthiest and safest possible manner.

## Introduction

Indisputable health improvement have occurred with the advent of antiretroviral therapy (ART), resulting in intense reductions in HIV-related morbidity and mortality and bring in improvements in quality of life [1, 2]. Breakthroughs in pregnancy care have reduced the risk of vertical transmission to 1 % if pregnant women receive timely ART, achieve optimal viral suppression, delivery by caesarean-section when appropriate, and avoid breastfeeding [3]. The availability of ART in poor setting changed the life persons with HIV from hopelessness to hopefulness and many of them decided to have children.

The need for an integrated approach to reproductive health and HIV was formally acknowledged as early as the 1994 International Conference on Population and Development and has since generated substantial policy support and academic interest [4].

In Sub-Saharan Africa (SSA), a region where reproductive- aged women account for the majority of people living with HIV [5], unintended pregnancies (unwanted or mistimed) are estimated to account for 14–58 % of all pregnancies in SSA [6]. The prevention of unintended pregnancy among women living with HIV has important implications for maternal and child health [7].

In countries like Ethiopia, where there is high prevalence of HIV, fertility patterns among persons living with HIV could have important role in HIV prevention and demographic effect.

Evidences documented so far on fertility desire among HIV infected clients receiving HAART have had variable results. For example, studies conducted in Africa including Ethiopia have shown that, the fertility desire after HAART initiation ranges from 31 – 60.9 % [8–10]. In Ethiopia, although few studies conducted in different health institutions, the information is limited and not recent regarding fertility desire and factors associated with it. In addition the extent of fertility desire among HIV infected clients receiving HAART and how these decisions may vary by individual, social, demographic characteristics and health factors is not well understood in the country. This research is therefore meant to assess level of fertility desire and factors associated with it among HIV infected clients receiving HAART at Finoteselam Hospital, Northwest Ethiopia. Accordingly, the findings of this research could be used as an input to safe mother hood programs at local and/or national levels.

## Methods

A cross sectional study supplemented by in-depth interview was conducted at Finoteselam Hospital, North West Ethiopia, from July – August, 2013. Finoteselam Town is found 375 km northwest from Addis Ababa, capital city of Ethiopia. In the town there is one district hospital, one health center, four private clinics and two health posts. The hospital has started providing ART services as payment free since 2005 and currently it has 3,543 HIV positive adults and adolescents in which 2,314 were on pre ART and 1,229 were actively following HAART. The hospital provides services for clients coming from all parts of the district and other neighboring health facilities.

The study population were those clients in reproductive age group (15-49 years old for women and 15-59 years old for men) receiving HAART at Finoteselam Hospital.

Totally 422 clients who fulfilled the inclusion criteria were included in the study by systematic random sampling technique. Three trained nurses who were working in ART clinics in other health facilities collected the data using structured questionnaire under daily supervision and follow up.

Data were entered in to EPI-Info version 3.5.1 then exported to SPSS version 16 for analysis. Descriptive and summary statistics were conducted. Proportion was calculated to estimate the fertility desire level. Univariate and multiple binary logistic regression models were fitted to identify factors associated with fertility desire (the need of clients on HAART to have child/children in their future life). Odds Ratio (OR) with 95 % confidence interval was computed and statistical significance was considered with P value less than 0.05.

To complement the quantitative study, in-depth interview was conducted with 11 key informants who were selected from clients and service providers purposely based on their socio-demographic characteristics like sex, marital status, and number of children they have. Each in-depth interview was carried out by principal investigators. It was tape recorded and field notes were taken. Then the entire audio taped interview was transcribed. The transcript then translated to English. The translated transcript was reviewed and examined line by line and highlighted using different colors by hand then categorized in to primary codes or themes. Later data was reviewed and combined in to broader concepts. The concepts were refined in to major themes.

Ethical clearance was obtained from the Research and Publications Committee of BahirDar University College of Medicine & Health Science and Gamby College of Medical Science. Formal letter of permission was obtained from Amhara National Regional State Health Bureau, and Finoteselam Hospital. Individual informed consent was obtained from respondents with age eighteen and above by explaining the purpose of the study clearly. For those with age less than eighteen years (three respondents) assent was obtained from themselves and consent was obtained from their parents. In order to keep confidentiality of study participants, names were not used and only those personnel trained on data collection and those who have prior experience were involved in the data collection and supervision process.

## Results

### Socio-demographic characteristics of study participants

A total of 422 eligible clients were participated in the study of which 217 (51.4 %) were males. The median age of participants was 33 (IQR = 12) years. The majority study participants 186 (44.1 %) were in age group of 30–39. More than half of the participants (64.7 %) lived in urban and married (70 %). With regard to participants’ educational status, 38.4 % of the study participants had no formal education (Table 1)

**Table 1:** Socio-demographic characteristics of clients on HAART at Finoteselam Hospital, Northwest Ethiopia July 2013

Variables		Numbers	Percent
Sex	Male	217	51.4
	Female	205	48.6
Age (years)	15–29	121	28.7
	30–39	186	44.1
	≥40	115	27.3
Residence	Urban	273	64.7
	Rural	149	35.3
Religion	Orthodox Christian	386	91.5
	Others	36	8.5
Educational status	No education	162	38.4
	Primary	18	4.3
	Secondary	150	35.5
	More than secondary	92	21.8
Marital status	Married	278	65.9
	Single	48	11.4
	Divorced/ Widowed	96	22.7
Ethnicity	Amhara	387	91.7
	Others	35	8.3
Occupational status	Employee	164	38.9
	Daily laborer	113	26.8
	Merchant	55	13.0
	House Wife	35	8.3
	Farmer	37	8.8
	Others	18	4.3
Monthly Income	<300 ETB	96	27.7
	301–600 ETB	164	38.9
	601–1000 ETB	73	17.3
	>1000 ETB	89	21.1

*ETB* Ethiopian Birr, 1ETB = 0.05 USD

.

### Child desire of study participants

Eighty two (37.8 %) of men and 59(28.8 %) of women, a total of 141(33.4 %) participants have desire for having children**.** A high desire for having children was seen in the age group 20–29 which was 51(42.1 %) followed by the age group of 30–39 which was 61(32.8 %). The reset 29 (25.2 %) desire were among age group ≥40 years. Among participants who had desire for having children, 68(48.2 %) need to have one child and 63(44.7 %) need to have two children, whereas 10(7.1 %) need three or more than three children. From participants who desire to have child/children 55(39.0 %) of them need to have child within one year, 54(38.3 %) within one to two years and 17(17.1 %) didn’t have specific time.

Those participants who have no desire for having children 281(66.6 %) had different reason for not desiring. More than half (53.7 %) of them responded that they have enough children, (36.7 %) had no enough money for new born child, 14 (5.0 %) of participants had fear of mother to child transmission of the virus, and the rest didn’t have desire for having children due to health service providers advice and had a fear of child bearing may further compromise their health condition.

The fertility history of participants showed 192 (45.5 %) of them had one or two child /children and 94(22.3 %) did not have children yet.; From the total of female respondents (205), 47(22.9 %) were pregnant in the study time, while 18(38.3 %) of pregnancy were unwanted or mistimed. Ninety seven (32.0 %) of participants partner want to have children in the future (Table 2)

**Table 2:** Reproductive history and Child desire of clients on HAART at Finoteselam Hospital, Northwest Ethiopia July 2013

Variables		Number	Percent
Current number of children			
	Have No child	94	22.3
	1–2	192	45.5
	≥3	136	32.2
Pregnancy now(*n* = 205)			
	Yes	47	22.9
	No	158	77.1
Pregnancy wanted/Timed (*n* = 47)			
	Yes	29	61.7
	No	18	38.3
Want children in the future(*n* = 422)			
	Yes	141	33.4
	No	281	66.6
Number of children wanted in the future (*n* = 141)			
	One	68	48.2
	Two	63	44.7
	More than two	10	7.1
Time prefer when to have children (*n* = 141)			
	Within 1 year	55	39.0
	Within 1 up to 2 years	54	38.3
	After 2 years	15	10.6
	Don’t Know specific time	17	12.1
Partner desire for having children (*n* = 304)			
	Yes	97	31.9
	No/ I don’t know	207	68.1

.

### Factors associated with fertility desire

In the multivariate binary logistic regression analysis Sex, Number of live children, Discussion with ART service provider (about their child desire, sexuality and family planning), and duration on ART were found significantly associated with fertility desire.

Being male was found to be positively associated with having fertility desire. Accordingly, male clients were 3.2 times more likely to have fertility desire compared to their female counterparts [AOR = 3.2, 95 % CI: (1.56, 6.51)]. Also, positive association was found between fertility desire and having no child. Clients who had no child were 6.7 times at higher desire for having child/children than those who had three or more children [AOR = 6.78, 95 % CI: (2.38, 19.27)]. In addition, having discussion with ART service provider with respect to their child desire, sexuality and family planning had shown direct association with increased fertility desire, that is, those clients who did have discussion with ART service provider had shown 3.1 times higher fertility desire compared to those clients who didn’t have discussion [AOR = 3.12, 95 % CI: (1.54, 6.32)]. On the other hand duration on ART follow up (in years) had shown indirect association with fertility desire. Being on ART follow up for less than or equal to 2 years had 3.6 times greater desire for fertility than being on the follow up for more than 2 years [AOR = 3.64, 95 % CI: (1.74, 7.64)] (Table 3)

**Table 3:** Logistic regression output for Fertility Desire among clients on HAART at Finoteselam Hospital, Northwest Ethiopia July, 2013

Variables	Fertility desire		OR (95 % CI)	
	YES	NO	Crude OR[95 % CI]	Adjusted OR [95 % CI]
Sex				
Female	59(28.8 %)	146(71.2 %)	1	1
Male	82(37.8 %)	135(62.2 %)	1.50 [1.00, 2.26]	3.18 [1.56, 6.51]^b^
Age				
>40 years	29(25.2 %)	86(74.8 %)	1	
15–29 years	51(42.1 %)	70(57.9 %)	2.16 [1.24, 3.76]^a^	1.97 [0.66, 5.87]
30–39 years	61(32.8 %)	125(67.2 %)	1.45 [0.86, 2.44]	1.36 [0.51, 3.60]
Monthly Income				
<300 ETB	37(38.5 %)	59(61.5 %)	1	
301–600 ETB	43(26.2 %)	121(73.8 %)	0.57 [0.33,0.971]^a^	0.43 [0.19, 1.01]
601–1000 ETB	27(37.0 %)	46(63.0 %)	0.94 [0.50, 1.75]	0.80 [0.25, 2.55]
>1000 ETB	34(38.2 %)	55(61.8 %)	0.99 [0.54, 1.78]	0.79 [0.27, 2.32]
Number of alive children				
≥ 3	20(14.7 %)	116(85.3 %)	1	1
1–2	70(36.5 %)	122(63.5 %)	3.33 [1.90, 5.82]^a^	2.22 [0.88, 5.58]
Have no child	51(54.3 %)	43(45.7 %)	6.88 [3.68, 12.84]^a^	6.78 [2.38, 19.27]^b^
Discuss with ART provider				
No	45(23.3 %)	148(76.7 %)	1	1
Yes	96(41.9 %)	133(58.1 %)	2.37 [1.55, 3.63]^a^	3.17 [1.54, 6.32]^b^
Duration on ART follow up				
> 2 years	75(29.1 %)	183(70.9 %)	1	1
≤ 2 years	66 (40.2 %)	98(59.8 %)	1.64 [1.09,2.48]^a^	3.64 [1.74, 7.64]^b^
Year since HIV Diagnosis				
>2 years	87 (30.2 %)	201(69.8 %)	1	
≤ 2 years	54(40.3 %)	80(59.7 %)	1.56 [1.02, 2.39]^a^	0.83 [0.21, 3.27]

^a^Significant association to Fertility Desire in univariate logistic regression analysis

*ETB* Ethiopian Birr, 1ETB = 0.05 USD

^b^Significant association to Fertility Desire in multiple logistic regression analysis

.

### Result of qualitative study

In the in-depth interview two service providers (one ART and one family planning) and nine clients were included as key informants (Table 4)

**Table 4:** Socio-demographic characteristics of in-depth interview participant clients on HAART at Finoteselam Hospital, Northwest Ethiopia July 2013

Characteristics		Number
**Age**		
	20–30	4
	31–40	3
	41–50	2
**Sex**		
	Female	5
	Male	4
**Marital status**		
	Married	7
	Single	1
	Divorced	1
**Number of children they have**		
	No child	3
	2 children	2
	3 children	1
	4 children	3

.

The in-depth interview of key informant clients had shown that the frequently expressed reason for having fertility desire were replacing themselves (two informants), to get someone to help in old ages (three informants), to make their family and community happy (two informants), to get means of reason to live and create happiness in family (one informant), to build self-esteem, cover their sero-status (HIV test result) in the community (two informant) and to give a sister for their daughter (one informant).

A 28 years old woman, who was married and had no child stated: *“… I need to have three children in the future. It helps me to replace myself*.” A 38 years old man, who was married and had four children described*: “…I need another two children a sister for my daughter.”* A 30 years old woman, who was married and had two children depicted*: “How can a woman live without having a number of children? I have two children, but I need three more children. That is how I get a reason to live and create happiness in my life.”*

A 41 years old man, who was married, an employee and had two children described*: “we need a child strongly. It gives us self-esteem, cover our HIV test result in the community and the child will take care of us in old age and children will take care of each other.”*

A 20 years old woman, who was single, sex worker and had no child said*:* “*I need at least one child who will take care of me and protect me from harm. Having children is important as one with no children will be forgotten when died*.”

ART service provider health professionals described: “*PLHIV who are on HAART has a great desire to have children. They want to replace themselves. Most of them frequently requests whether they can give birth or not and push us to allow them. Especially those who had one or no child are so eager. They want to have negative child despite possibility of mother to child transmission. We always told them not to become pregnant for the sake of their health. We counsel all the alternatives and leave decision for them.”*

Reasons for not having fertility desire expressed by the key informants were age, having children and economical problem.

## Discussion

The availability of ART and effective prevention of mother to child transmission makes fertility desire a great reproductive health issue for persons living with HIV. This study tried to assess the level of fertility desire and identify factors associated with it.

Fertility desire of clients on HAART at Finoteselam Hospital was found to be 33.4 %. This result was relatively consistent with a study conducted in Hosanna, South Ethiopia, in which there was 36.5 % proportion of fertility desire [11], but higher than a study conducted in Northeastern Ethiopia 18.3 % [12]. In contrast, the level of fertility desire in the current study was slightly lower than a study conducted in Northwest of the country, at Gondar University hospital which found 42.16 % of participants had a desire to have a child / children. Similarly, the current finding was lower than the finding of a study conducted in the Eastern part of Ethiopia which was 56.2 % [13, 14]. Also similar differences were seen in comparison to a South African study done in Cape Town in which 49 % of participants had a desire for child/children [10].

Generally, the level of fertility desire in the current study was lower compared to previous studies. This might be due to time difference and better health service providers counseling about the effects of having child on their health and possibility of mother to child transmission in the current time. This might decreased the level of desire to have child/children. The other possible explanation can be the decrease in level of stigma and discrimination towards people living with HIV in the current time. This go in line with the in-depth interview, informants who desire to have child/children stated that having child/children will hade there status so that the stigma and discrimination towards them will decrease.

The current study had shown that male clients (37.8 %) had higher desire than females (28.8 %). This finding was similar with a study conducted in Nekemte (West Ethiopia) which found 40.5 % of men and 30.7 % of women had fertility desire [15]. Also similar results were found in studies conducted in Northern and Southern parts of Ethiopia [11, 14]. In addition to this fertility desire difference between male and female clients was similarly higher among males in a study conducted in South Africa [10]. The possible explanation might be that females may fear for their health condition that it can be devastated during pregnancy and child birth. Thus, they might have lower desire of fertility compared to males.

In the multiple logistic regression analysis current number of children was significantly associated with fertility desire. Participants who had no child were 6.7 times more likely to have desire for children than those who had three or more children. This finding was in line with the findings of studies conducted in Western, Northwestern and Southern Ethiopia and also with South African study [10, 11, 14, 15]. This may be due to the reason that those who did not have child need to replace themselves and as in culture of developing countries, they need someone to taker of them when they get old and weak. This was supported by the in-depth interview in the way that informants desire to have child/children to replace them and to have someone in their old age taking care of them.

Those clients who did have discussion with ART service provider were 3.1 times at higher desire for having child/children compared to those who didn’t have discussion. This finding may show that ART service provider give advice to clients that promote fertility but the possible explanation for this association may be that those clients who had fertility desire seek advice from service provider and have discussion. This was supported by the in-depth interview of the ART service provider. The service provider stated that clients having desire to have child/children seek advice about it and try to push the service provider to approve their desire. Thus, having desire might make them to have discussion but not the other way round.

In this study duration on ART (in years) was also independently associated with fertility desire. Clients who had been on ART less or equal to two years were 3.6 times more likely to have a desire for having children than those who had been more than two years on ART. This finding was similar with the association seen in a study conducted in Nekemte (Western Ethiopia). This could be due to the reason that access to ART would improve their health status in the first years of follow up compared to their pre-ART status. This wellbeing feeling, in turn, may lead them to desire for having child/children.

The strength of this study was that it was complemented with the qualitative exploration of factors.

## Conclusions

One third of participants have desire to have a child/children in the future. Being male, having no child, less or equal to two years on ART and seeking discussion with ART service provider were found to be associated with higher desire of fertility. Since significant number of ART clients have desire to have a child/children in the future, guidelines formulated and counseling protocols developed shall consider this to achieve their reproductive goals in the healthiest and safest possible manner. Providers of ART service shall continue on counseling ART clients about their fertility desire and family planning utilization to optimize healthy outcomes for mother, father, and baby.

## Abbreviations

AOR: Adjusted odds ratio
ART: Antiretroviral treatment
HAART: Highly active antiretroviral treatment
HIV: Human immunodeficiency virus
IQR: Inter quartile range
OR: Odds ratio
SPSS: Statistical package for social sciences
SSA: Sub-Saharan Africa
